# Pentraxin-3 as a novel prognostic biomarker in non-neutropenic invasive pulmonary aspergillosis patients

**DOI:** 10.1128/spectrum.02945-24

**Published:** 2025-01-29

**Authors:** Chao Sun, Xiaomin Cai, Huanhuan Zhong, Yajie Lu, Yuanyuan Li, Yuchen Cai, Yujie Wang, Tingting Zhao, Min Cao, Li Wang, Chunlai Feng, Wenkui Sun, Cheng Chen, Yujian Tao, Guoer Ma, Binchan He, Xinyu Wang, Jinjin Zhong, Xin Lu, Yuanqin Li, Xin Su

**Affiliations:** 1 Department of Respiratory and Critical Medicine, Nanjing Drum Tower Hospital, Affiliated Hospital of Medical School, Nanjing University, Nanjing, China; 2 Department of Respiratory and Critical Medicine, Jinling Hospital, Affiliated Hospital of Medical School, Nanjing University, Nanjing, China; 3 Department of Respiratory and Critical Medicine, The Second Affiliated Hospital of Soochow University, Suzhou, China; 4 Department of Respiratory and Critical Medicine, Nanjing First Hospital 385685 , Nanjing, China; 5 Department of Respiratory and Critical Medicine, Changzhou First People’s Hospital, Changzhou, China; 6 Department of Respiratory and Critical Medicine, Jiangsu Province Hospital 74734 , Nanjing, China; 7 Department of Respiratory and Critical Medicine, The First Affiliated Hospital of Soochow University, Suzhou, China; 8 Department of Respiratory and Critical Medicine, Affiliated Hospital of Yangzhou University 632468 , Yangzhou, China; 9 Department of Respiratory and Critical Medicine, Affiliated Hospital of Jiangsu University 191612 , Zhenjiang, China; 10 Department of Respiratory and Critical Medicine, Jiangsu Province Second Chinese Medicine Hospital, Nanjing, China; 11 Department of Respiratory and Critical Medicine, Nanjing Jiangning Hospital, Nanjing, China; 12 Department of Respiratory and Critical Medicine, Affiliated Hospital of Xuzhou Medical University, Xuzhou, China; Central Texas Veterans Health Care System, Temple, Texas, USA

**Keywords:** invasive pulmonary aspergillosis, prognostic factors, pentraxin-3, mycological biomarkers

## Abstract

The incidence of invasive pulmonary aspergillosis (IPA) in non-neutropenic patients is increasing. This study aimed to evaluate the clinical outcomes and risk factors for mortality in non-neutropenic IPA patients. We conducted a prospective, multicenter study from August 2020 to February 2024, enrolling 565 patients with suspected IPA. The study cohort comprised 195 IPA (non-neutropenic) cases and 370 non-IPA cases. Peripheral blood and bronchoalveolar lavage fluid (BALF) specimens were collected to measure pentraxin-3 (PTX3) levels. Additionally, demographic data, clinical characteristics, and antifungal therapy of each patient were recorded. We analyzed factors associated with 30- and 90-day mortality. IPA patients exhibited higher mortality rates compared to non-IPA patients, with 30-day rates of 26.15% versus 8.38% (*P* < 0.001) and 90-day rates of 34.36% versus 13.24% (*P* < 0.001). Higher plasma and BALF PTX3 levels were associated with poor prognosis in IPA patients. ROC curve analysis identified optimal PTX3 thresholds of 4.29 ng/mL in BALF (sensitivity, 67.1%; specificity, 81.4%) and 7.11 ng/mL in plasma (sensitivity, 73.4%; specificity, 82.8%) for predicting mortality. Multivariate Cox regression analysis confirmed PTX3 levels in plasma (hazard ratio [HR] 3.87, 95%CI [1.87–8.00], *P*＜0.001) and BALF (HR 2.40 [1.19–4.84], *P* = 0.014) were independent prognostic factors for IPA mortality. Additionally, positive galactomannan test results in both BALF and plasma were initially correlated with increased mortality in IPA patients. However, after adjusting for potential confounding factors, this correlation no longer remained statistically significant. In conclusion, PTX3 is a promising prognostic biomarker of mortality in IPA patients.

**IMPORTANCE:**

Studies have confirmed the value of pentraxin-3 (PTX3) in the diagnosis of invasive pulmonary aspergillosis (IPA), yet its prognostic significance in IPA remains unclear. This study found that in non-neutropenic IPA patients, plasma and bronchoalveolar lavage fluid (BALF) levels of PTX3 are independently associated with poor outcomes. Furthermore, the optimal cutoff values of PTX3 for predicting a poor prognosis of IPA are 4.29 ng/mL in BALF and 7.11 ng/mL in plasma. These findings could help us better manage IPA in non-neutropenic patients, potentially enhancing the prognosis of patients with this condition.

## INTRODUCTION

Invasive pulmonary aspergillosis (IPA) is a fungal infection caused by *Aspergillus*, typically affecting individuals with immune dysfunction. From 2010 to 2023, there has been a notable increase in the global incidence of invasive aspergillosis, with an estimated 2.113 million new cases each year. This rise is largely attributed to the increasing prevalence of chronic obstructive pulmonary disease (COPD), diabetes, critical illness, and lung cancer. Despite advancements in antifungal therapies, the annual mortality remains high at 85.2% (1). The high mortality is largely attributed to delayed diagnosis and treatment, as well as the absence of effective risk assessment tools necessary for clinical decision-making.

The non-specific clinical and radiological features of non-neutropenic IPA pose diagnostic challenges, underscoring the need for robust biomarkers. Recent guidelines recommend the use of *Aspergillus* culture, galactomannan (GM) testing, and *Aspergillus* PCR testing as essential components of the diagnostic process (2, 3). Previous studies have also shown that the level of *Aspergillus*-specific IgG (*Asp* IgG) in plasma was valuable for diagnosing IPA in non-neutropenic patients (4). These biomarker levels assist in assessing the activity of *Aspergillus* infection and reflect immune response. Notably, among COVID-19 patients, those exhibiting three or more positive mycologic criteria showed significantly higher mortality rates (5). This finding emphasizes the critical role of mycological biomarkers in prognostic monitoring. However, their prognostic value in non-neutropenic IPA remains uncertain.

Pentraxin 3 (PTX3) is a pattern recognition receptor. Our prior work has demonstrated that PTX3 enhances the phagocytosis of *Aspergillus* spores by macrophages, thereby facilitating fungal clearance (6). PTX3 is primarily produced by monocytes and macrophages, and its levels significantly increase in response to infection and inflammation. Current studies are investigating the role of PTX3 in pulmonary aspergillosis. Notably, certain PTX3 single nucleotide gene types have been linked to an increased susceptibility to IPA in non-neutropenic patients (7, 8). Furthermore, plasma and bronchoalveolar lavage fluid (BALF) PTX3 levels were significantly higher in IPA patients compared to those with other types of pathogen infections or without infection (9, 10). Meanwhile, persistently elevated PTX3 levels may exacerbate inflammation and tissue damage. High PTX3 levels were associated with poor prognosis in various diseases (11, 12). However, the role of PTX3 in predicting the prognosis of non-neutropenic IPA patients remains unexplored.

In this study, we aim to determine whether plasma and BALF PTX3 levels can serve as prognostic biomarkers in non-neutropenic IPA patients by investigating the clinical outcomes and risk factors. We found that compared to traditional inflammatory markers and mycological biomarkers, plasma and BALF PTX3 levels serve as robust prognostic biomarkers in non-neutropenic IPA patients.

## MATERIALS AND METHODS

### Study design

We performed a multicenter, prospective cohort study across 12 hospitals from Jiangsu province from August 2020 to February 2024. Non-neutropenic patients suspected of IPA were included in this study. “Suspected IPA” was defined by the following criteria: (i) main host factors, including but not limited to, chronic lung diseases and immunosuppressive conditions; (ii) at least one respiratory infection sign or symptom, such as cough, fever, hemoptysis, and chest pain, with no response to empirical antibiotic therapy; (iii) abnormal chest CT features, such as consolidation or infiltrates. The exclusion criteria included (i) neutropenic patients (peripheral blood neutrophil count＜0.5 × 10^9^/L) during hospitalization; (ii) *Aspergillus* colonization, chronic pulmonary aspergillosis (CPA), and allergic bronchopulmonary aspergillosis (ABPA); (iii) without a definitive diagnosis; and (iv) clinical information deficiency.

### Diagnostic criteria of IPA

The grading diagnostic criteria of IPA referred to the 2020 guidelines from the European Organization for Research and Treatment of Cancer and the Mycoses Study Group Education and Research Consortium (MSGERC) (2), and the 2024 consensus definitions from ESGCIP, EFISG, ESICM, ECMM, MSGERC, ISAC, and ISHAM (3). “Proven IPA” requires histopathologic confirmation of *Aspergillus* hyphae in a normally sterile site or the lung obtained through biopsy or needle aspiration. “Probable IPA” needs a comprehensive evaluation of host factors, symptoms, clinical criteria, and mycological evidence. Host factors and symptoms were described above. The clinical criterion is a chest CT scan showing infiltrates or cavitation not attributable to other causes. Mycological evidence included one or more of the following: a serum or plasma GM level greater than 0.5 optical density index (ODI), a BALF GM level at least 1.0 ODI, or positive *Aspergillus* culture from BALF, bronchial brush, or aspirate, and at least two positive *Aspergillus* PCR tests. “Possible IPA” met host factors, clinical symptoms, and clinical criteria. In our cohort, all patients categorized as “possible IPA” showed positive findings on next-generation sequencing (NGS) of respiratory tract specimens and had a favorable response to anti-*Aspergillus* therapy.

### Patients and samples

**Fig 1:**
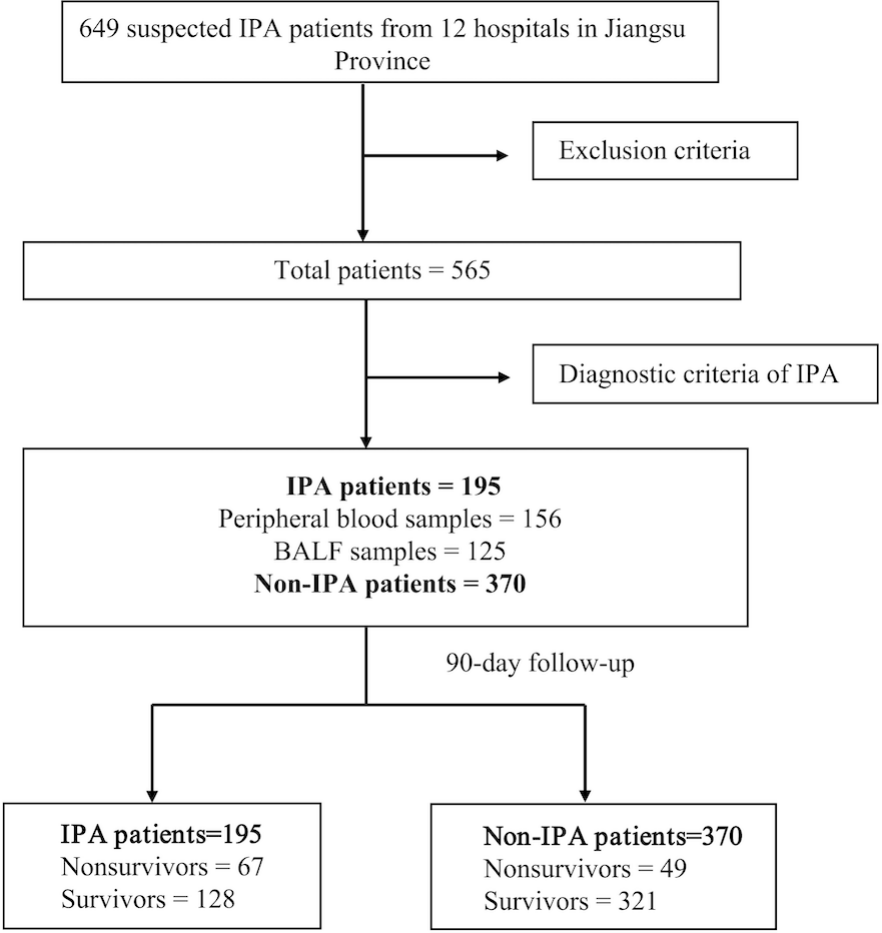
The flow chat in this study.

Our study initially enrolled 649 patients with suspected IPA. After excluding 84 patients (2 cases of neutropenic, 13 cases with incomplete clinical information, 7 cases with unclear diagnosis, 28 cases of CPA, 7 cases of ABPA, 5 cases of *Aspergillus* colonization, and 22 cases post-antifungal treatment), 565 patients remained for analysis. Of these, 195 patients were diagnosed with IPA and 370 patients were non-IPA. Among the 370 non-IPA patients, there were 259 cases of pulmonary infection, 38 cases of tuberculosis, 20 cases of lung cancer, 10 cases of lung abscess, 20 cases of interstitial lung disease, and 23 cases of non-infectious diseases. Each patient provided at least one sample before antifungal therapy, either from peripheral blood or BALF. A total of 125 BALF and 156 peripheral blood samples were collected. After centrifugation, plasma and BALF supernatant were stored at −80℃. The flowchart is presented in Fig. 1.

### PTX3 test, *Asp* IgG test, data collection, and outcome

Levels of PTX3 in BALF and plasma were determined utilizing ELISA kit (DPTX30, Quantikine Human Pentraxin 3 Immunoassay, R&D, USA) according to the provided protocol. Additionally, the levels of *Asp*-specific IgG in plasma were quantified with the *Aspergillus* IgG Quantitative Test Kit (Dynamiker, China). Additional data, including demographics, comorbidities, clinical features, laboratory tests, and imaging features at admission, as well as treatment information, were collected from electronic health records. The primary endpoints were 30 and 90 days of death. The secondary outcomes were intensive care unit (ICU) admission and death in the ICU. Survival data were obtained through follow-up phone calls.

### Group stratification based on PTX3 levels

IPA patients were categorized into three groups based on their PTX3 levels. The distribution of PTX3 levels was divided into tertiles, with the upper tertile classified as the high-level group, the middle tertile as the medium-level group, and the lower tertile as the low-level group.

### Statistics

Statistical analysis was conducted using SPSS (version 29.0, IBM Corporation, NY, USA). Continuous variables were presented as mean ± standard deviation (SD) or median (interquartile range [IQR]), while categorical variables were summarized as counts (percentages). Comparative analyses were performed using the Chi-square test, Fisher’s exact test, Student’s *t* test, or Mann-Whitney *U* test, as appropriate. Receiver operating characteristic (ROC) curve analysis was used to determine the sensitivity, specificity, and optimal threshold. Cox regression analysis was conducted to estimate the hazard ratio (HR) and 95% confidence interval (CI) for 90-day mortality. Variables with univariate analysis *P* < 0.05 were included in multivariate analysis. Input method was used to perform regression analysis. Ninety-day survival data were analyzed using the Kaplan-Meier method with the log-rank test for comparisons. In order to assess the survival at different time points, we also employed the landmark analysis method. Statistical significance was defined as *P* < 0.05, using two-tailed hypothesis tests.

## RESULTS

### Clinical outcomes of all patients in our study

In the cohort, a total of 195 patients with IPA and 370 without IPA were enrolled. The 30-day all-cause mortality rate among IPA patients was 26.15% (51/195), and the all-cause 90-day mortality rate was 34.36% (67/195), which were significantly higher than those of non-IPA patients (30 days: 51/195 [26.15%] vs 31/370 [8.38%], *P* < 0.001; 90 days: 67/195 [34.36%] vs 49/370 [13.24%], *P* < 0.001). Additionally, IPA patients had longer hospital stays (15 [10, 24] vs 10 [7, 15], *P*＜0.001) and higher ICU admission rates (88/195 [45.13%] vs 66/370 [17.84%], *P* < 0.001) (Table S1).

### Clinical characteristics of IPA patients in our study

**Fig 2:**
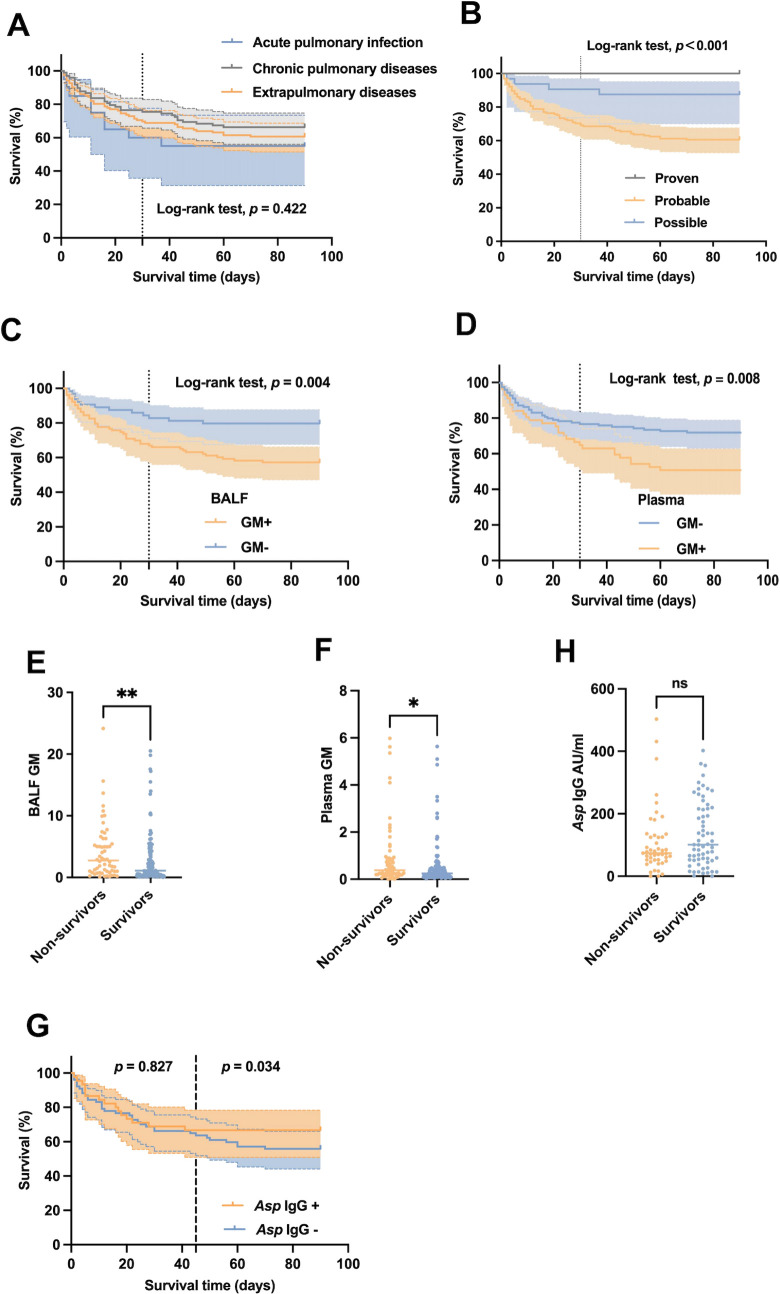
The relationship between host factors, mycological evidence, and prognosis of IPA patients. (**A–D and G**) Kaplan-Meier survival curve compares the relationship between 90-day mortality of IPA patients and host factors, diagnostic criteria, BALF GM, plasma GM, and Asp IgG results. Statistical significance was assessed with log-rank test. Survival analysis at different time periods using landmark analysis. (**E and F**) BALF and plasma GM levels in the non-survival group and survival group. (**H**) Plasma Asp IgG level in the non-survival group and survival group. Statistical significance was assessed with Wilcoxon-Mann-Whitney test. **P < 0.01 and *P < 0.05.*Asp*, *Aspergillus*.

The clinical characteristics between survivors and non-survivors of IPA are detailed in Table 1. Nearly all patients (98.46%, 192/195) had at least one host factor, most commonly diabetes mellitus (25.64%, 50/195) and COPD (25.64%, 50/195). The 90-day mortality rates for IPA patients with acute viral infections, chronic lung diseases, and extrapulmonary diseases were 45.00% (9/20), 33.63% (38/113), and 34.68% (43/124), respectively. Kaplan-Meier survival analysis revealed no statistically significant differences in the cumulative survival rates among the three groups (*P* = 0.422) (Fig. 2A). The highest mortality rate was observed in cases of COVID-19-associated pulmonary aspergillosis (CAPA), reaching 60.00% (6/10). Additionally, non-survivors had a higher prevalence of cardiovascular comorbidities (*P* < 0.001).

**TABLE 1:** The clinical characteristics between the survivors and non-survivors of IPA^*e*^

Variables	Total (*n* = 195)	Non-survivors (*n* = 67)	Survivors (*n* = 128)	*P* value
Baseline characteristics				
Age (years), median (IQR)	68 (57, 75)	72 (64, 78)	66 (56, 73)	<0.001
Male, *n* (%)	152 (77.95)	52 (77.61)	100 (78.13)	0.934
BMI (kg/m^2^), median (IQR)	21.66 (19.44, 23.67)	20.99 (18.71, 23.59)	21.87 (19.52, 23.88)	0.327
Host factors, *n* (%)				
Acute viral infection				
COVID-19	10 (5.13)	6 (8.96)	4 (3.13)	0.095
Influenza A and B	10 (5.13)	3 (4.48)	7 (5.47)	0.999
Chronic lung diseases				
COPD	50 (25.64)	15 (22.39)	35 (27.34)	0.452
Lung cancer	27 (13.85)	13 (19.40)	14 (10.94)	0.104
Bronchiectasis	36 (18.46)	10 (14.93)	26 (20.31)	0.357
Extrapulmonary diseases				
Solid tumor	34 (17.44)	13 (19.40)	21 (16.41)	0.600
Renal translation	6 (3.08)	0	6 (4.69)	0.096
Diabetes	50 (25.64)	19 (28.36)	31 (24.22)	0.530
Blood system diseases	10 (5.13)	1 (1.49)	9 (7.03)	0.169
Autoimmune disease	24 (12.31)	10 (14.93)	14 (10.94)	0.421
Comorbidities				
Cardiovascular disease	83 (42.56)	40 (59.70)	43 (33.59)	<0.001
Medical history				
Systemic corticosteroids^*b*^	34 (17.44)	11 (16.42)	23 (17.97)	0.271
Inhaled corticosteroids^*c*^	18 (9.23)	3 (4.48)	15 (11.72)	1.121
Immunosuppressants^*d*^	16 (8.21)	6 (8.96)	10 (7.81)	0.276
Clinical Symptoms, *n* (%)				
Cough	176 (90.26)	59 (88.06)	117 (91.41)	0.456
Sputum	167 (85.64)	58 (86.57)	109 (85.16)	0.834
Dyspnea	119 (61.03)	48 (71.64)	71 (55.47)	0.028
Fever^*a*^	115 (58.97)	47 (70.15)	68 (53.13)	0.022
Hemoptysis	34 (17.44)	7 (10.45)	27 (21.09)	0.075
Chest CT Features, *n* (%)				
Nodules				
1–3 cm in diameter	47 (24.10)	15 (22.39)	32 (25.00)	0.686
<1 cm in diameter	56 (28.72)	15 (22.39)	41 (32.03)	0.158
Consolidation or infiltration	133 (68.21)	42 (62.69)	91 (71.09)	0.231
Pleural effusion	85 (43.59)	48 (71.64)	37 (28.91)	<0.001
Cavitation	58 (29.74)	14 (20.90)	44 (34.38)	0.050
Air-crescent sign	7 (3.59)	1 (1.49)	6 (4.69)	0.426
Tree-in-bud pattern	13 (6.67)	2 (2.99)	11 (8.59)	0.225
Lesion distribution pattern				
Multiple	158 (81.03)	58 (86.57)	100 (78.13)	0.181
Bilateral diffuse	16 (8.21)	7 (10.45)	9 (7.03)	0.421
Laboratory test, median (IQR)				
WBC (×10^9^/L)	9…50 (6…37, 13.14)	10.88 (8.14, 14.74)	8.40 (6.10, 12.26)	0.002
Neutrophil (×10^9^/L)	7.28 (4.32, 10.86)	9.58 (7.11, 12.78)	6.27 (4.109, 10.18)	<0.001
CRP (mg/L)	57.80 (13.10, 121.30)	109.40 (55.40, 160.30)	40.60 (5.57, 78.60)	<0.001
PCT (ng/mL)	0.18 (0.08, 0.65)	0.47 (0.18, 1.41)	0.13 (0.05, 0.35)	<0.001
PLT (×10^9^/L)	203.50 (160.75, 269.25)	192.00 (134.00, 253.75)	209.50 (169.25, 277.50)	<0.001
ALB (g/L)	30.70 (26.65, 35.70)	27.45 (25.20, 32.48)	32.10 (28.00, 37.30)	<0.001
Creatinine (μmol/L)	63.90 (50.00–90.60)	68.85 (50.75, 118.88)	62.30 (50.00, 81.50)	0.142
Co-infection, *n* (%)				
Bacteria	54 (27.69)	26 (38.81)	28 (21.88)	0.012
Fungi	12 (6.15)	6 (8.06)	6 (4.69)	0.239
Mycobacterium	10 (5.13)	6 (8.06)	4 (3.13)	0.095
Severity				
ICU admission, *n* (%)	88 (45.13)	51 (76.12)	37 (28.91)	<0.001
PSI, media (IQR)	100 (75, 135)	144 (121, 175)	85 (66, 107)	<0.001
Mycological criteria positive/performed, *n* (%)				
BALF culture	34/155 (21.94)	12/54 (22.22)	22/101 (21.78)	0.950
Serum GM	57/182 (31.82)	28/63 (44.44)	29/119 (24.37)	0.006
BALF GM	103/167 (61.68)	44/57 (77.19)	59/110 (53.64)	0.003
BALF NGS	109/131 (83.21)	29/37 (78.38)	70/94 (74.47)	0.639
Serum *Asp* IgG	43/108 (39.81)	15/45 (33.33)	28/63 (44.44)	0.245

^*a*^ Temperature exceeding 37.3°C.

^*b*^ Oral or intravenous glucocorticoids were used for more than 3 weeks within a 60-day period.

^*c*^ Inhalation corticosteroids were used for more than 3 weeks within a 60-day period.

^*d*^ Immunosuppressive drugs were used within a 30-day period.

^*e*^ BMI, body mass index; CT, computed tomography; WBC, white blood cell; CRP, C-reactive protein; PCT, procalcitonin; PLT, platelet; ALB, albumin; PSI, pneumonia severity index; and Asp, *Aspergillus*.

A total of 191 patients received antifungal treatment, with voriconazole being the predominant choice. One hundred twenty-seven patients with IPA received voriconazole monotherapy, with a 30-day mortality rate of 18.90% (24/127) and a 90-day mortality rate of 26.77% (34/127). Additionally, 52 patients underwent combination therapy, which included voriconazole, caspofungin, and/or amphotericin B, resulting in a 30-day mortality rate of 46.15% (24/52) and a 90-day mortality rate of 53.85% (28/52). Combined therapy mainly occurs in critically ill patients (Table S2). Notably, IPA patients who had a favorable 90-day prognosis experienced a shorter interval from hospital admission to the initiation of antifungal treatment compared to those who did not survive (4 [2–6] vs 5 [3–10], *P* = 0.024).

The average age of IPA patients was 66 years, with non-survivors being older (72 vs 66, *P* < 0.001). Non-survivors also presented more severe clinical conditions, including higher ICU admission rates (76.12% vs 28.91%, *P* < 0.001) and elevated pneumonia severity index (PSI) scores (144 vs 85, *P* < 0.001). Symptoms such as fever (70.15% vs 53.13%, *P* = 0.022) and dyspnea (71.64% vs 55.47%, *P* = 0.028) were more frequent in this group, and pleural effusion was markedly more common (71.64% vs 28.91%, *P* < 0.001). Furthermore, non-survivors showed elevated levels of white blood cells (WBCs), neutrophils, platelets, C-reactive protein (CRP), and procalcitonin (PCT) (all *P* < 0.05), while serum albumin levels were higher in survivors (*P* < 0.001). Bacterial co-infections were identified in 54 IPA patients, with *Pseudomonas aeruginosa* being the most common pathogen (12/54), followed by *Acinetobacter baumannii* (11/54). These infections were more prevalent in non-survivors (38.81% vs 21.88%, *P* = 0.012).

### Prognostic value of mycological biomarkers in IPA patients

Based on the diagnostic criteria, the cohort of 195 IPA patients was classified into 13 proven cases, 133 probable cases, and 49 possible cases. During the 90-day follow-up, no deaths occurred in the proven IPA group. However, Kaplan-Meier survival analysis indicated that the 90-day mortality in the probable IPA group was substantially higher than that in the possible IPA group (44.36% vs 16.33%, *P* < 0.001) (Fig. 2B). Subsequently, we explored the prognostic value of various mycological tests, including BALF culture, BALF and plasma GM assays, BALF NGS, and plasma *Asp* IgG levels. Detailed results of these mycological examinations are presented in Table 1.

The positivity rate of GM test was 61.68% (103/167) in BALF and 31.82% (57/182) in plasma. Among GM-positive patients, the 90-day mortality rate was significantly higher than in GM-negative patients for both BALF (43.72% vs 20.31%, *P* = 0.003) and plasma (49.12% vs 28.00%, *P* = 0.006). The cumulative survival rate in the GM-positive group was significantly reduced (Fig. 2C and D). Additionally, GM levels were higher in non-survivors compared to survivors in both BALF (2.88 [1.00, 5.44] ODI vs 1.07 [0.40, 3.75] ODI, *P* = 0.004) and plasma (0.38 [0.20, 0.88] ODI vs 0.25 [0.13, 0.49] ODI, *P* = 0.017) (Fig. 2E and F). Among 108 patients who underwent *Asp* IgG testing, 43 tested positive (value above 120 AU/mL) with a mortality rate of 34.88% (15/43), while 65 patients with negative results had a mortality rate of 46.15% (30/65), showing no significant difference in 90-day mortality (*P* = 0.245). However, Kaplan-Meier analysis found that positive *Asp* IgG levels were associated with lower mortality of IPA patients within 45–90 days (*P* = 0.034) (Fig. 2G). *Asp* IgG level did not significantly differ between non-survivors and survivors (73.82 [52.29, 128.15] AU/mL vs 100.94 [51.79, 214.46] AU/mL, *P* = 0.202) (Fig. 2H). Additionally, no correlation was found between the positive results of BALF culture and BALF NGS and the prognosis of IPA (Table 1).

### Independent predictors of mortality in IPA patients

The optimal cutoff values for continuous variables were determined using ROC curve analysis (Table S3). Multivariate Cox regression analysis revealed that admission to the ICU (HR = 3.06, 95% CI = 1.10–8.52, *P* = 0.033), PSI score exceeding 114 (HR = 3.05, 95% CI = 1.16–7.98, *P* = 0.023), and presence of pleural effusion (HR = 3.06, 95% CI = 1.42–6.59, *P* = 0.004) are independent prognostic factors for 90-day mortality in IPA patients (Table 2).

**TABLE 2:** Factors associated with 90-day mortality in IPA patients: multivariate Cox regression analysis^*a*^

Variables	HR	95% CI	*P* value
Age (≥71 years)	1.02	0.48–2.19	0.954
Cardiovascular disease	0.69	0.33–1.44	0.317
Combination bacterial infection	0.77	0.36–1.62	0.484
Fever	1.05	0.44–2.50	0.914
Dyspnea	1.95	0.89–4.30	0.098
Plasma-GM (≥0.52 ODI)	0.97	0.44–2.11	0.928
BALF-GM (≥2.60 ODI)	1.64	0.84–3.19	0.145
WBC (≥7.51 × 10^9^/L)	0.81	0.13–5.27	0.829
Neutrophil (≥6.37 × 10^9^/L)	1.37	0.21–8.91	0.742
PLT (≥151 × 10^9^/L)	0.50	0.22–1.10	0.086
CRP (≥80.15 mg/L)	1.53	0.75–3.09	0.242
PCT (≥0.16 ng/mL)	1.00	0.40–2.51	0.998
ALB (≥29.25 g/L)	0.75	0.38–1.50	0.416
Pleural effusion	3.06	1.42–6.59	0.004
PSI (≥114 score)	3.05	1.16–7.98	0.023
ICU admission	3.06	1.10–8.52	0.033

^*a*^ CT, computed tomography; PLT, platelet; ALB, albumin; and PSI, pneumonia severity index.

### Distribution of serum and BALF PTX3 in IPA patients

**Fig 3:**
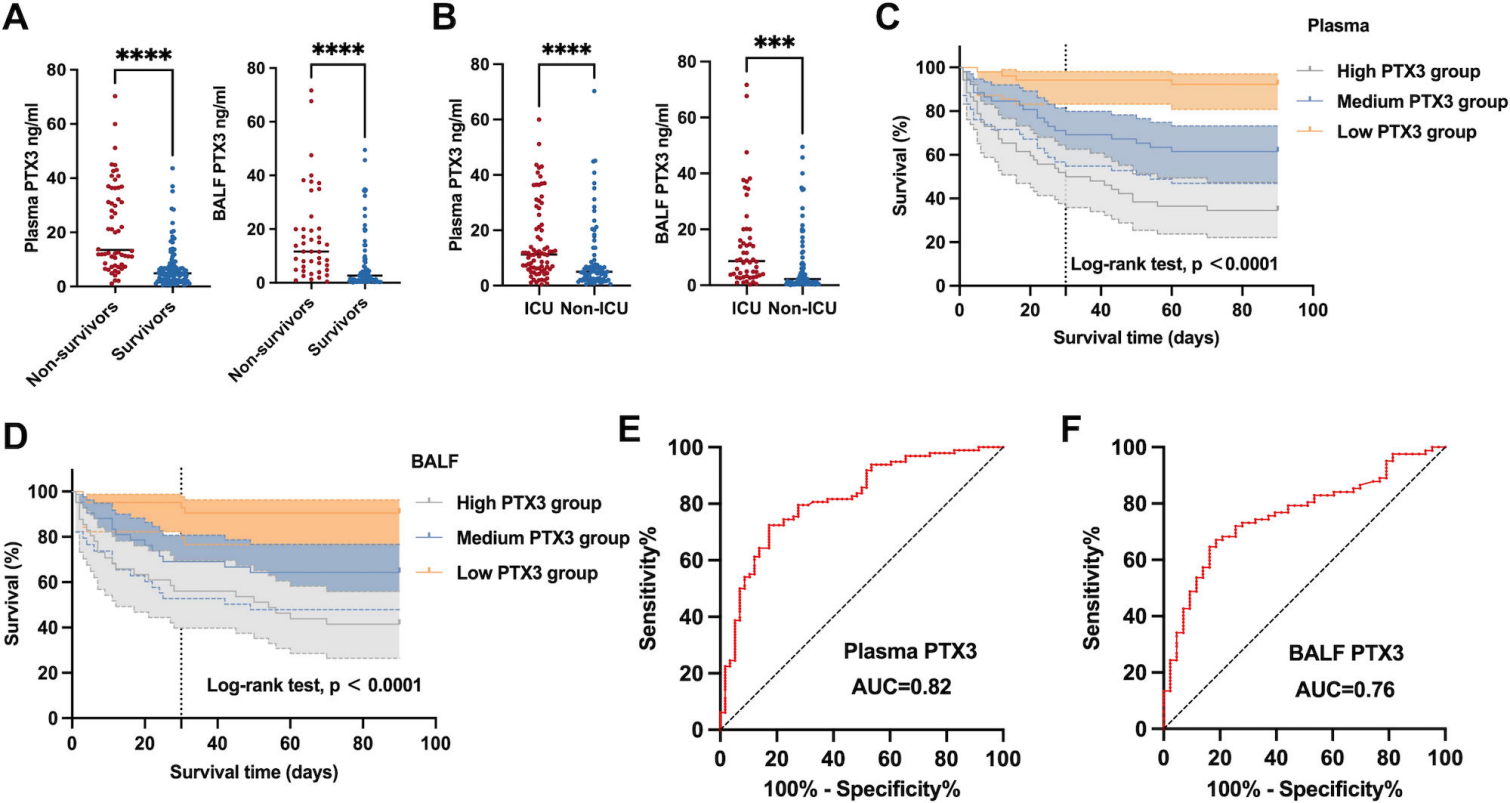
Prognostic potential of PTX3 levels in IPA patients. (**A and B**) BALF and plasma PTX3 levels in non-survival and survival groups and ICU and non-ICU groups. Statistical significance was assessed with Wilcoxon-Mann-Whitney test. ***P < 0.001 and ****P < 0.0001. (**C and D**) Kaplan-Meier survival curve compares the relationship between 90-day mortality of IPA patients and the high, medium, and low PTX3 levels. High group: 11.90–70.30 ng/mL in plasma and 9.23–71.7 ng/mL in BALF; medium group: 4.76–11.9 ng/mL in plasma and 2.16–9.23 ng/mL in BALF; and low group: 0.43–4.76 ng/mL in plasma and 0.09–2.16 ng/mL in BALF. Statistical significance was assessed with log-rank test. (**E and F**) ROC analysis of PTX3 levels in plasma and BALF for the identification of 90-day mortality in IPA patients.AUC, area under the curve.

A total of 156 plasma and 125 BALF specimens from IPA patients were analyzed for PTX3 levels. In line with previous findings that established correlations between PTX3 and conventional inflammatory markers (8), our study also found positive correlations between PTX3 levels and CRP and PCT levels in both plasma and BALF (Table S4). PTX3 levels were significantly elevated in non-survivors compared to survivors in both plasma (13.50 [8.24, 35.32] ng/mL vs 4.91 [2.32, 8.25] ng/mL, *P* < 0.0001) and BALF (11.66 [5.44, 20.09] ng/mL vs 2.68 [0.97, 8.02] ng/mL, *P* < 0.0001) (Fig. 3A). Moreover, ICU patients also had higher PTX3 levels than those in general wards (11.27 [6.04, 21.80] ng/mL vs 5.02 [2.32, 9.09] ng/mL, *P* < 0.0001 in plasma, 8.67 [3.51, 17.71] ng/mL vs 2.21 [0.90, 11.22] ng/mL, *P* < 0.001 in BALF) (Fig. 3B). We classified the IPA patients into three groups based on their PTX3 levels. IPA patients were categorized into high (11.90–70.30 ng/mL in plasma and 9.23–71.7 ng/mL in BALF), medium (4.76–11.9 ng/mL in plasma and 2.16–9.23 ng/mL in BALF), and low (0.43–4.76 ng/mL in plasma and 0.09–2.16 ng/mL in BALF) PTX3 groups. Kaplan-Meier survival analysis demonstrated significantly lower survival rates in the high PTX3 groups for both plasma and BALF (*P* < 0.0001 for both) (Fig. 3C and D).

### Prognostic value of PTX3 levels in IPA patients

ROC curve analysis was used to determine the PTX3 thresholds for differentiating non-survivors from survivors. In plasma, a PTX3 level above 7.11 ng/mL yielded an AUC of 0.82 (95%CI 0.75–0.89), with a sensitivity of 82.8% and specificity of 73.4% (Fig. 3E). In BALF, a PTX3 level greater than 4.29 ng/mL resulted in an AUC of 0.76 (95%CI 0.68–0.85), with a sensitivity of 81.4% and specificity of 67.1% (Fig. 3F).

To assess the prognostic significance of PTX3, we performed multivariate Cox regression analyses on IPA patients with collected plasma and BALF samples, respectively. Elevated PTX3 levels in both plasma and BALF were identified as independent predictors of adverse outcomes in IPA (Table 3). Notably, PTX3 levels in plasma demonstrated superior predictive accuracy compared to BALF (plasma: aHR = 3.87, 95% CI = 1.87–8.00, *P* < 0.001; BALF: aHR = 2.40, 95% CI = 1.19–4.84, *P* = 0.014). These findings underscore the potential of PTX3 as a biomarker for enhancing the predictive accuracy of clinical outcomes in IPA.

**TABLE 3:** Multivariate Cox regression analysis of the prognostic value of PTX3 in plasma and BALF

Variables	HR	95% CI	*P* value
Plasma (*n* = 156)			
Plasma-PTX3 (≥7.11 ng/mL)	3.87	1.87–8.00	<0.001
PSI (≥114 score)	4.44	1.87–10.54	<0.001
Pleural effusion	1.95	1.06–3.60	0.032
ICU admission	1.56	0.77–3.14	0.215
BALF (*n* = 125)			
BALF-PTX3 (≥4.29 ng/mL)	2.40	1.19–4.84	0.014
PSI (≥114 score)	2.21	1.01–4.81	0.046
Pleural effusion	2.09	0.97–4.53	0.061
ICU admission	3.15	1.45–6.85	0.004

## DISCUSSION

Previous research on the prognosis of IPA has primarily focused on immunocompromised patients, with non-neutropenic cases neglected to subgroup. Our study represents the largest prospective cohort to date, elucidating outcomes among non-neutropenic IPA patients while providing a comprehensive assessment of mortality risk factors. We found that IPA patients exhibit higher mortality compared to non-IPA. Furthermore, our results demonstrate that PTX3 levels in both plasma and BALF were independent predictors of 90-day mortality in IPA patients.

The mortality of IPA was influenced by immune status and underlying diseases (13). Neutropenic IPA patients exhibit higher mortality, with 90-day mortality in those with hematological malignancies reaching up to 75.2% (14). Conversely, the 90-day mortality of non-neutropenic patients has been reported to range from 18.1% to 44.8% (15, 16), consistent with our findings. In this study, IPA patients with a favorable 90-day prognosis had a shorter interval between hospital admission and the initiation of antifungal therapy, emphasizing the critical role of timely intervention in improving IPA outcomes.

PTX3 is not only a soluble pattern recognition receptor but also serves as an inflammatory cytokine. Elevated PTX3 levels reflect a worse inflammatory response. The prognostic value of PTX3 has been assessed across various diseases, including COVID-19, lung cancer, vasculitis, and sepsis (11, 12, 17–19). However, no previous studies have systematically assessed the relationship between PTX3 levels and the severity or prognosis of IPA. Traditional inflammatory markers, such as CRP, have been linked to poor outcomes in IPA patients (20, 21). Notably, in the context of COVID-19, PTX3 has emerged as a strong predictor of short-term mortality, outperforming conventional markers (11). Our findings in non-neutropenic IPA patients support this conclusion. The association between elevated PTX3 levels and adverse outcomes suggests that an inflammatory storm may be a key factor driving poor short-term prognosis in IPA patients. This underscores the potential of PTX3 not only as a diagnostic tool but also as a marker for monitoring disease progression in IPA.

The radiological characteristics of non-neutropenic IPA patients lack specificity. The incidence of pleural effusion in IPA varies considerably across studies (20, 22, 23). Similarly, the prognostic value of pleural effusion remains inconsistent. While previous studies have linked pleural effusion to adverse outcomes in IPA (20, 24), Park et al*.* (25) found no correlation between pleural effusion and higher mortality rates in both neutropenic and non-neutropenic IPA patients. PSI is a tool for assessing the severity and prognosis of community-acquired pneumonia (26). In this study, PSI scores were found to be correlated with the prognosis of non-neutropenic IPA patients. Previous research has demonstrated the effectiveness of PSI in evaluating the prognosis of hospital-acquired pneumonia, highlighting its utility in assessing the severity of pulmonary infections and predicting outcomes (27).

While positive mycological test results have been associated with the prognosis of IPA, a clear consensus across different studies remains elusive. In immunocompromised IPA patients, dynamic monitoring of GM levels is particularly important for prognostic assessment (28, 29). The prognostic value of GM in non-neutropenic IPA patients remains unclear, with several recent studies investigating its role in CAPA prognosis. Bartoletti et al. (30) found that higher initial BALF-GM levels are independently associated with an increased 30-day mortality rate. However, Hurt et al*.* (31) found no correlation between BALF-GM levels and 90-day outcomes in CAPA patients. Additionally, *Asp* IgG typically emerges after a period of infection, with significant elevation observed in patients with a disease course exceeding 10 days (32). Bergeron et al*.* (28) found no correlation between baseline antibody levels and the 45-day prognosis of IPA patients, which is consistent with our findings; however, we identified a correlation between baseline *Asp* IgG levels of ≥120 AU/mL and survival between 45 and 90 days. Future studies should investigate the role of the dynamic changes in *Asp* IgG in the prognosis of IPA patients.

This study has several limitations in assessing the clinical characteristics and prognosis of IPA patients. First, the study primarily focuses on the short-term prognosis of non-neutropenic IPA patients, neglecting the long-term impact of microbiological indicators and PTX3 levels. Additionally, the study excludes severely immunocompromised neutropenic patients, who have a higher mortality rate, leaving the prognostic value of PTX3 in this population unexplored. Finally, the study lacks a validation cohort, necessitating larger future cohorts to confirm the prognostic value of PTX3.

### Conclusion

In conclusion, this study provides new insights into the clinical management of IPA in non-neutropenic patients. Our findings highlight that PTX3 levels, rather than traditional mycological biomarkers, play a crucial role in determining the 90-day prognosis of IPA. Elevated PTX3 levels in both BALF and plasma emerged as independent predictors of poor prognosis. Furthermore, we observed that the time from hospital admission to the initiation of antifungal therapy was significantly longer in the non-survivor group. These results suggest that an exaggerated inflammatory response and delayed antifungal treatment are critical risk factors contributing to poor outcomes in IPA patients.

## SUPPLEMENTAL MATERIAL

Supplemental tables spectrum.02945-24-s0001.docx Tables S1 to S4.

ASM does not own the copyrights to Supplemental Material that may be linked to, or accessed through, an article. The authors have granted ASM a non-exclusive, world-wide license to publish the Supplemental Material files. Please contact the corresponding author directly for reuse.
