# Supplementary material for: Pentraxin-3 as a novel prognostic biomarker in non-neutropenic invasive pulmonary aspergillosis patients
Source: Microbiol Spectr. 2025 Jan 29;13(3):e02945-24. doi: 10.1128/spectrum.02945-24 (PMC11878064; doi:10.1128/spectrum.02945-24)
Supplement: Supplemental tables — Tables S1 to S4. [file spectrum.02945-24-s0001.docx]

**Supplementary**

Tabel S1 The clinical outcomes of IPA and non-IPA patients.

|  | **IPA (n=195)** | **Non-IPA (n=370)** | **P value** |
| --- | --- | --- | --- |
| 30-day all-cause mortality, n (%) | 51 (26.15) | 31 (8.38) | ＜0.001 |
| 90-day all-cause mortality, n (%) | 67 (34.36) | 49 (13.24) | ＜0.001 |
| Hospital stay, media [IQR] | 15 [10, 24] | 10 [7,15] | ＜0.001 |
| ICU admission, n (%) | 88 (45.13) | 66 (17.84) | ＜0.001 |

IPA, invasive pulmonary aspergillosis; IQR, interquartile range; ICU, intensive care unit.

Table S2 The comparison of severity between monotherapy and combination therapy for IPA patients.

| **Variables** | | **Monotherapy (n=127)** | **Combination therapy (n=52)** | **P value** |
| --- | --- | --- | --- | --- |
| Age, year, media [IQR] | 67 [56-74] | | 69 [60-75] | 0.22 |
| ICU admission, n (%) | 49 (38.58） | | 28 (53.85) | 0.06 |
| PSI, media [IQR] | 89 [68-124] | | 122 [97-166] | ＜0.001 |
| Plasma-PTX3, ng/ml, media [IQR] | 6.20 [2.33-12.94] | | 11.43 [5.22-22.91] | 0.004 |
| BALF-PTX3, ng/ml, media [IQR] | 3.81 [1.18-14.02] | | 6.00 [2.21-16.94] | 0.31 |

IPA, invasive pulmonary aspergillosis; IQR, interquartile range; ICU, intensive care unit; BALF, bronchoalveolar lavage fluid; PTX3, pentraxin 3.

Tabel S3 The optimal cutoff values of continuous variables.

| **Variables** | **Cut-off value** | **AUC (95%CI)** | **Sensitivity%** | **Specificity%** |
| --- | --- | --- | --- | --- |
| Age, year | 71.5 | 0.65 (0.57-0.73) | 50.7 | 71.9 |
| WBC (×10^9^/L) | 7.51 | 0.64 (0.55-0.72) | 80.6 | 44.8 |
| Neutrophil (×10^9^/L) | 6.37 | 0.68 (0.60-0.76) | 82.3 | 51.2 |
| CRP, mg/L | 80.15 | 0.74 (0.66-0.81) | 66.2 | 75.8 |
| PCT, ng/ml | 0.16 | 0.74 (0.67-0.82) | 82.0 | 58.3 |
| PLT (×10^9^/L) | 151 | 0.41 (0.33-0.50) | 66.7 | 16.9 |
| ALB, g/L | 29.25 | 0.30 (0.22-0.38) | 35.5 | 32.2 |
| Plasma-GM, ODI | 0.52 | 0.60 (0.52-0.69) | 42.9 | 79.3 |
| BALF-GM, ODI | 2.60 | 0.64 (0.55-0.72) | 52.6 | 71.3 |
| PSI, score | 114 | 0.86 (0.80-0.92) | 80.6 | 79.7 |

BALF, bronchoalveolar lavage fluid; WBC, white blood cell; CRP, C-reactive protein; PCT, procalcitonin; PLT, platelet; ALB, albumin; PSI, pneumonia severity index; GM, galactomannan; ODI, optical density index.

Table S4 The correlation between PTX3 and inflammatory markers.

| **Variables** | **R** | **P value** |
| --- | --- | --- |
| **Plasma-PTX3, ng/ml** |  |  |
| WBC (×10^9^/L) | 0.40 | ＜0.001 |
| Neutrophil (×10^9^/L) | 0.48 | ＜0.001 |
| CRP, mg/L | 0.56 | ＜0.001 |
| PCT, ng/ml | 0.44 | ＜0.001 |
| **BALF-PTX3, ng/ml** |  |  |
| WBC (×10^9^/L) | 0.32 | ＜0.001 |
| Neutrophil (×10^9^/L) | 0.36 | ＜0.001 |
| CRP, mg/L | 0.19 | 0.03 |
| PCT, ng/ml | 0.38 | ＜0.001 |

BALF, bronchoalveolar lavage fluid; PTX3, pentraxin 3; WBC, white blood cell; CRP, C-reactive protein; PCT, procalcitonin.
